# Decreased Efficiency of Between-Network Dynamics During Early Memory Consolidation With Aging

**DOI:** 10.3389/fnagi.2022.780630

**Published:** 2022-05-16

**Authors:** Ronja V. Faßbender, Okka J. Risius, Julian Dronse, Nils Richter, Hannes Gramespacher, Qumars Befahr, Gereon R. Fink, Juraj Kukolja, Oezguer A. Onur

**Affiliations:** ^1^Cognitive Neuroscience, Institute of Neuroscience and Medicine (INM-3), Research Center Jülich, Jülich, Germany; ^2^Department of Neurology, Faculty of Medicine and University Hospital Cologne, University of Cologne, Cologne, Germany; ^3^Department of Neurology and Clinical Neurophysiology, Helios University Hospital Wuppertal, Wuppertal, Germany; ^4^Faculty of Health, Witten/Herdecke University, Witten, Germany

**Keywords:** episodic memory, consolidation, aging, resting-state fMRI, fALFF, networks, ventral visual stream, DMN

## Abstract

Aging is associated with memory decline and progressive disabilities in the activities of daily living. These deficits have a significant impact on the quality of life of the aging population and lead to a tremendous burden on societies and health care systems. Understanding the mechanisms underlying aging-related memory decline is likely to inform the development of compensatory strategies promoting independence in old age. Research on aging-related memory decline has mainly focused on encoding and retrieval. However, some findings suggest that memory deficits may at least partly be due to impaired consolidation. To date, it remains elusive whether aging-related memory decline results from defective consolidation. This study examined age effects on consolidation-related neural mechanisms and their susceptibility to interference using functional magnetic resonance imaging data from 13 younger (20–30 years, 8 female) and 16 older (49–75 years, 5 female) healthy participants. fMRI was performed before and during a memory paradigm comprised of encoding, consolidation, and retrieval phases. Consolidation was variously challenged: (1) control (no manipulation), (2) interference (repeated stimulus presentation with interfering information), and (3) reminder condition (repeated presentation without interfering information). We analyzed the fractional amplitude of low-frequency fluctuations (fALFF) to compare brain activity changes from pre- to post-encoding rest. In the control condition, fALFF was decreased in the left supramarginal gyrus, right middle temporal gyrus, and left precuneus but increased in parts of the occipital and inferior temporal cortex. Connectivity analyses between fALFF-derived seeds and network ROIs revealed an aging-related decrease in the efficiency of functional connectivity (FC) within the ventral stream network and between salience, default mode, and central executive networks during consolidation. Moreover, our results indicate increased interference susceptibility in older individuals with dynamics between salience and default mode networks as a neurophysiological correlate. Conclusively, aging-related memory decline is partly caused by inefficient consolidation. Memory consolidation requires a complex interplay between large-scale brain networks, which qualitatively decreases with age.

## Introduction

Aging is associated with progressive cognitive decline and impairment in the activities of daily living (Christensen et al., 2009; Deborah and Smeeding, 2010). These deficits have a significant impact on the quality of life of the aging population and lead to a tremendous burden on societies and health care systems (Barberger-Gateau and Fabrigoule, 1997; Black and Rush, 2002; Christensen et al., 2009; Deborah and Smeeding, 2010). Age-related cognitive decline is predominant in memory compared to other cognitive abilities (Schönknecht et al., 2005; Grady, 2008). However, several studies have shown that memory is not uniformly affected by age. Episodic memory is particularly vulnerable to the effects of aging (Brickman and Stern, 2009). It is essential to learn and store new information to perform the activities of daily living. Understanding which mechanisms lead to episodic memory decline can help us develop compensatory strategies and thus promote independence in old age.

There has been considerable research on aging-related decline in episodic memory and its neural substrates, mainly focusing on encoding- or retrieval-related processes. A meta-analysis by Spreng et al. (2010) confirmed increased activity during encoding in the right middle frontal gyrus, medial temporal lobes, and right putamen and decreased activity in the right precentral sulcus for younger compared to older individuals. For memory retrieval, an increased activity in the posterior occipital fissure and a decreased activity in the rostrolateral prefrontal cortex, the supplementary motor area, and the middle temporal gyrus were confirmed for younger compared to older individuals. Since many of the areas with age differences were part of the so-called ‘task-positive network’ (TPN), the authors assume an age-dependent recruitment of the TPN during memory encoding and retrieval. Thus, aging-related memory decline seems to be not only explained by activity changes in individual brain regions, but, by changes in large-scale brain networks. Further studies support this assumption. A study examining functional connectivity (FC) within the default mode network (DMN) found reduced FC between the anterior and posterior components of the DMN with increasing age using correlation analyses over the time courses within *a priori* defined regions (Andrews-Hanna et al., 2007). Also, the structural integrity within DMN and salience network (SN) is impaired in terms of reduced cortical thickness with increasing age. Superagers with preserved memory exhibit significantly less atrophy in regions of the DMN and SN compared to older individuals with memory deficits (Sun et al., 2016).

However, recent studies suggest that deficits in episodic memory are not only due to deficient neural processes during encoding and retrieval but at least partly due to impaired memory consolidation (Craig et al., 2016a). *Memory consolidation* is defined as the “progressive post-acquisition stabilization of memory” that begins immediately after new information is encoded and continues for many hours or days. According to the standard model of memory consolidation, a memory trace becomes more and more stable over time, transferring the encoded information into long-term memory (Dudai, 2004). During consolidation, however, the memory trace is still vulnerable to retroactive interference, i.e., during this phase, new sensory input can overwrite what has been learned. Retroactive interference explains, for example, the phenomenon of memorizing your new phone number and not being able to recall your old number after some time. Thereby, the effect of retroactive interference is greater the more similar the learned material and the interfering material are. The robust finding that retrieval performance decreases upon interference with consolidation processes supports the assumption that memory deficits are in part due to impaired memory consolidation and not exclusively due to encoding or retrieval deficits (Dewar et al., 2007, 2012).

Nevertheless, it remains controversial whether aging-associated memory decline can be explained by aging-related deficits in consolidation processes. Craig et al. (2016b) propose two possible mechanisms how memory consolidation might be affected by aging: (1) Memory consolidation processes become less efficient with increasing age due to qualitatively or quantitatively reduced neural activity, and (2) become more susceptible to interference with increasing age.

Investigating the first hypothesis is challenging as consolidation, unlike encoding and retrieval, is unrelated to a conscious experience or specific cognitive function. Therefore, it is difficult to assess the processes involved in consolidation. To date, there is no experimental paradigm that directly measures consolidation, neither behaviorally nor neurophysiologically.

Two forms of consolidation are distinguished, fast synaptic consolidation and slow systems consolidation. The latter leads to changes in the systems or brain circuits involved in encoding. In this process, the memory trace is reorganized, shifting the burden of storage from the hippocampus, which was particularly involved in encoding the information, to the neocortex. Thus, the neocortex can independently retain and retrieve the memory trace (Dudai, 2004).

Traditionally, sleep is thought to be necessary for this process (Euston et al., 2007; Girardeau et al., 2009). During sleep, individuals remain undisturbed by any external sensory input, which creates an optimal setting for the reactivation of neural activity beneficial for the gradual transmission of the memory trace from the hippocampus to the neocortex. The assumption that sleep plays an important role in memory consolidation is supported by studies examining the effects of sleep disorders on memory performance. For example, a meta-analysis on studies with older adults showed that self-reported poor sleep correlated with impairments in several cognitive domains, including memory (Lo et al., 2016). Objective measures of sleep also show that disturbed sleep is related to impairments in memory consolidation and thereby memory performance. Mander et al. (2013) showed that age-related changes in slow-wave activity during sleep above the prefrontal cortex are associated with increased hippocampal activation during a retrieval task the next day. Thus, the memory trace has not become independent of the hippocampus, indicating deficient consolidation. This finding implies that the effect of deficient consolidation on age-related memory decline is mediated by increasingly poor sleep in old age (Mander et al., 2017).

However, sleep seems to be beneficial but not crucial for memory consolidation. There is growing evidence that system consolidation already occurs immediately after encoding (Tambini and Davachi, 2019). Thus, to examine the effect of age on memory consolidation without the influence of age-related sleep disturbances, we focus on consolidation during the awake resting state in the present study. Moreover, in an experimental setting, it can be ensured that individuals remain undisturbed by any external sensory input similar to sleep. Therefore, the analysis of blood oxygenation level-dependent (BOLD) resting-state functional magnetic resonance imaging (fMRI) during wakeful rest after encoding is a promising technique to investigate the neural mechanisms involved in system consolidation and the effect of aging thereon.

Recent resting-state (rs) fMRI studies have shown that activation patterns initially evident during encoding spontaneously reoccur during post-encoding rest and sleep (Deuker et al., 2013). Moreover, FC of local brain areas active during encoding and those active during retrieval increases from pre- to post-encoding rest (Risius et al., 2019), likewise hippocampal-vmPFC crosstalk increases during consolidation (Van Kesteren et al., 2010). In line with the standard model of memory consolidation and the multitrace theory (Nadel et al., 2007), these studies indicate that during consolidation the memory trace is stabilized through the replay of neural activity and is distributed across different brain regions. Accordingly, it is reasonable to assume that different memory-associated brain regions cooperate in networks during memory consolidation. A network or whole-brain rather than a ROI-based approach is required to verify this idea. To examine the effect of age on the neural mechanisms during consolidation Kukolja et al. (2016) used such a network-based approach and found that subsequent memory performance was predicted by increased FC in a temporo-occipital network and decreased FC in the default mode network during the resting phase after encoding. Older compared to younger individuals failed to show increased connectivity in the right lingual gyrus as part of an extended default mode network during the consolidation phase. Additionally, Jacobs et al. (2015) analyzed within-network FC and the orchestration of different memory-associated large-scale brain networks during post-encoding rest. They found higher competition between the default mode and the executive network during post-encoding rest, which was associated with better retrieval performance in older compared to younger individuals.

Age differences in the neural mechanisms underlying consolidation were only addressed in the latter two studies reported. However, these studies cannot answer the question of an age-dependent effect of interference on neural consolidation processes because either no interference condition was implemented (Jacobs et al., 2015) or they were not designed to measure the effect of interference at the stimulus level (Kukolja et al., 2016). The present study fills this gap by implementing an interference condition that induces stimulus-specific interference of the information being consolidated.

Nevertheless, there are already a number of behavioral studies on the effect of interference on subsequent memory performance, with contradictory results (e.g., Craig et al., 2016b; Martini et al., 2019). However, as age-related decline in executive functions such as inhibition capacity has been frequently reported in the literature (Gunning-Dixon and Raz, 2003; Resnick et al., 2003; Kramer et al., 2005; Allain et al., 2007), we expect that older individuals have a reduced ability to suppress new spatial information from the interference task, resulting in overwriting of the spatial position learned during encoding. Accordingly, we expect that the consolidation of the spatial information learned during encoding will be impaired, leading to deficient memory performance. In the present study, we introduce a more sophisticated measure of interference susceptibility than retrieval performance after interference, which has been used in most studies on this topic (Dewar et al., 2007, 2012; Craig et al., 2016b; Muecke et al., 2018). In the present study, interference-related errors are differentiated from general errors and related to successful retrieval of the material to be remembered. We propose this measure as a more sensitive indicator of interference susceptibility during consolidation.

Finally, besides interference with consolidation processes, we are interested in whether experimentally induced memory reactivation through exposure to cues for recently learned information can strengthen consolidation processes in older adults. A central mechanism of consolidation seems to be the repeated reactivation of activity patterns from encoding. Previous studies in rodents (Dupret et al., 2010) and humans have already shown that the number of spontaneous reactivations during consolidation can predict memory performance (Deuker et al., 2013). Furthermore, the presentation of cues for previously learned information during sleep, during a working-memory task (Oudiette et al., 2013) and during wakeful rest (Alm et al., 2019) can induce a reactivation of activity patterns from encoding, resulting in improved subsequent memory performance. Another study in which the learned information was presented again for a very brief period during a cover task also showed a beneficial effect on subsequent memory performance. This effect was stronger the worse the performance in a previous immediate recall task was (Tambini et al., 2017). Accordingly, we expect that especially older individuals will benefit from targeted memory reactivation and that this could represent a potential compensatory strategy for age-related memory decline.

Taken together, the present study investigates whether aging-related memory decline can be explained by mechanisms of memory consolidation. We hypothesize that memory consolidation processes become less efficient with increasing age due to qualitatively or quantitatively reduced neural activity and functional connectivity (H1). Besides, we hypothesize that susceptibility to interference increases with age, as indicated by changes in neural activity and functional connectivity during consolidation (H2). Finally, we assume that experimentally induced repeated reactivation of information to be remembered supports consolidation processes and thus may serve as a compensatory strategy for aging-related memory decline due to impaired consolidation processes (H3).

## Materials and Methods

### Sample

A total of 36 cognitively healthy participants attended the experiment and gave written informed consent in accordance with the Declaration of World Medical Association of Helsinki (2001). Participants were recruited as part of the AMATE study (Aging and MCI and Alteration of Top-Down Control during Episodic Memory Encoding) through advertisements. Participants had no contraindication for MRI measurements, no psychiatric or neurological disorders, and were not on medication acting on the central nervous system. All participants were native German speakers, had normal or corrected to normal vision, and were right-handed, as assessed by the Edinburgh handedness inventory (Oldfield, 1971). According to our inclusion criteria, we recruited individuals between 18 and 30 years of age for healthy young individuals and between 50 and 89 years of age for healthy senior individuals. Groups differed significantly in years of age and education. According to Fisher’s Exact Test, gender was equally distributed in both groups (Table 1).

**TABLE 1:** Group comparison of demographic variables.

	HY M(SD)	HS M(SD)	*t*-value	df	*p*-value
years of age	24.46 (2.85)	65.62 (8.03)	19.98^a^	19.42^a^	<0.001^a^
years of education	17.77 (2.74)	14.25 (3.11)	3.19	27	0.004
female | male	8 | 5	5 | 11			0.14^b^

*^a^adjusted for the violation of the assumption of equal variances.*

*^b^p-value of Fisher’s Exact Test.*

*HY, healthy young; HS, healthy seniors; df, degrees of freedom.*

Participants had to be excluded from further analyses due to (1) substantial signal dropout due to technical difficulties (*N* = 1), (2) memory performance below the probability of guessing (*N* = 1) (3) substantial deviation from the study protocol (e.g., no filler task during the consolidation session; *N* = 1), (4) extensive head-motion exceeding 3mm head translations in any direction (Johnstone et al., 2006) (*N* = 2), and (5) subject-specific mean motion (FD Jenkinson; Framewise Displacement as per Jenkinson et al., 2002) exceeding the group mean by more than three times the interquartile range (outlier detection; *N* = 2). Thus, 29 participants entered further analyses with 13 healthy young (HY) and 16 healthy senior (HS) participants.

A comprehensive neuropsychological test battery was applied before the fMRI experiment covering memory, attention, visuospatial abilities, language, and executive functions. Neuropsychological testing was performed as part of the AMATE study, as Risius et al. (2019) described. Based on the neuropsychological test results and questionnaires, all participants were classified as cognitively normal and without depressive and psychiatric symptoms by an experienced neuropsychologist. As expected, groups differed significantly in neuropsychological tests concerning episodic memory and executive functions (Table 2).

**TABLE 2:** Group comparison of neuropsychological test results.

	HY M(SD)	HS M(SD)	*t*-value	df	*p*-value	*95% CI* ^b^	
verbal memory – immediate recall (VLMT)	66 (4.34)	56.69 (9.26)	2.19	19	0.041*	3.26	14.63
verbal memory – delayed recall (VLMT)	14.31 (1.18)	12.06 (2.86)	2.92^a^	18.08^a^	0.009**^a^	0.70	4.09
verbal memory – recognition (VLMT)	14.85 (0.38)	13.51 (2.19)	2.62^a^	14^a^	0.02*^a^	0.54	2.73
visual memory – immediate recall (WMS)	80.69 (10.98)	62.63 (16.02)	2.74	19	0.013*	3.54	33.56
visual memory – delayed recall (WMS)	73.92 (12.43)	57.71 (15.32)	3.13	19	0.006**	6.97	37.30
visual memory – recognition (WMS)	17.31 (3.35)	16.27 (2.17)	1.10	19	0.29	−3.13	4.87
visual working memory (symbol span)	30.62 (6.81)	24.56 (5.48)	2.00	19	0.06	−0.60	10.39
auditory short-term memory (digit span)	6.92 (1.26)	6.5 (1.37)	1.39	19	0.18	−0.50	2.32
auditory working memory (digit span)	6.08 (1.44)	5.31 (0.95)	1.12^a^	6.33^a^	0.31^a^	−0.55	2.21
psychomotor speed (TMT-A in seconds)	18.92 (12.2)	32.81 (15.83)	−4.07^a^	15.66^a^	0.001**^a^	−26.37	−9.75
cognitive flexibility (TMT-B/TMT-A)	2.47 (0.89)	2.12 (0.56)	1.58	19	0.13	−0.07	0.97
executive functions (Stroop in seconds)	65.54 (8.55)	84.88 (16.62)	−2.40	19	0.027*	−29.79	−7.37
divided attention (BTA)	17.83 (2.64)	18.8 (1.32)	−1.13	19	0.27	−3.83	1.10
mental rotation (LPS 7)	25.83 (9.35)	15.25 (7.82)	2.65	19	0.016*	1.14	19.02
semantic verbal fluency (RWT)	57.77 (8.59)	45.88 (9.93)	2.18	19	0.042*	2.63	18.29
phonemic verbal fluency (RWT)	35.85 (7.2)	30.38 (8.61)	0.29	19	0.77	−5.79	8.67

*Differences in neuropsychological test results between healthy young individuals (HY) and healthy seniors (HS).*

*^a^adjusted for the violation of the assumption of equal variances.*

*^b^bias-corrected and accelerated (BCa) bootstrapping with 5000 iterations.*

HY, healthy young group; HS, healthy senior group; VLMT, Verbal Learning and Memory Test; WMS, Wechsler Memory Scale; TMT, Trail Making Test; BTA, Brief Test of Attention; LPS, Leistungsprüfsystem (performance test system); RWT, Regensburger Word Fluency Test. *p < 0.05; **p < 0.01; *** p < 0.001.

### Experimental Design

The present study represents a mixed design. Both, the participants in the HS group and the participants in the HY group completed three fMRI experiments approximately 1 week apart (*Mode* = 7 days). Participants performed a spatial contextual memory task in each experiment, consisting of an encoding, a subsequent consolidation, and a retrieval phase. The consolidation phase was interrupted by a second divergent task to induce a modulatory effect on memory consolidation. The three fMRI experiments varied in the extent to which consolidation processes were modulated. The fMRI experiment with the control task (no interference with previously encoded information) is further referred to as the *control condition (CON).* The fMRI experiment with the reminder task (repeated presentation of the stimulus *without* the interference of spatial context information) is called the *reminder condition (RE)*, and the fMRI experiment with the interference task (repeated presentation of the stimulus *with* interference of spatial context information) is further referred to as the *interference condition* (*INT*, for more details, see section Stimuli and Task). The order of the three conditions was randomized to account for sequence effects (Supplementary Table 1). Randomization of the order of conditions was statistically tested and confirmed after exclusion of outliers using Fisher’s exact test (*p* = 0.989).

**FIGURE 1:**
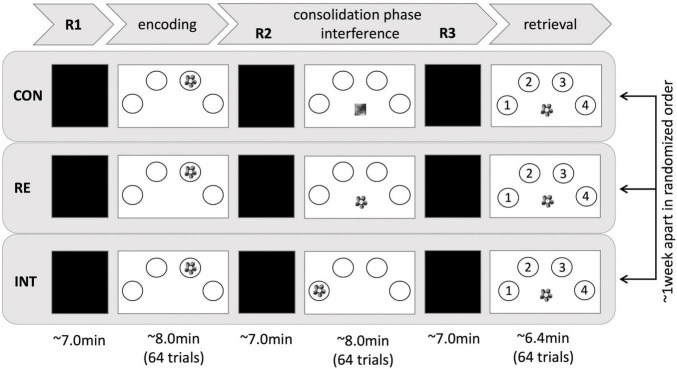
Experimental setup. Three fMRI sessions, differing in the type of interference, took place 1 week apart and in randomized order. In each session, a contextual memory task was implemented, consisting of an encoding, a consolidation, and a retrieval phase. Resting periods occurred before encoding (R1), after encoding (R2), and before retrieval (R3). CON, control condition; RE, reminder condition; INT, interference condition.

Additionally, three resting-state fMRI (rsfMRI) measurements were obtained in each experiment: a baseline rsfMRI at the beginning of the experiment (pre-encoding rest; R1), a rsfMRI after the initial encoding task (immediate post-encoding rest; R2), and a rsfMRI after the modulatory task (delayed post-encoding rest; R3; Figure 1). Participants were instructed to keep their eyes closed and relax without falling asleep during the acquisition.

### Stimuli and Task

The spatial contextual memory task used in the present study is an adaptation of a well-established paradigm (Cansino et al., 2002; Kukolja et al., 2009, 2016), consisting of an encoding, a modulating, and a retrieval task. The encoding task consisted of 64 trials. During each trial, participants saw a stimulus, showing a colored photograph of a natural or artificial (man-made) object. The stimuli were randomly presented in one of four circles on a white screen, arranged in a horizontal semi-circle bent to the top. Participants had to memorize the object and its respective position. To ensure alertness, participants had to indicate whether the object was natural or artificial by pressing two corresponding keys with their right index and middle finger, respectively. Each stimulus was presented for 3 s with a variable inter-trial interval of 2 to 12 s, followed by a fixation cross presented for 2 s.

The consolidation phase was interrupted by a modulatory task that differed between conditions in the extent to which consolidation processes were supposed to be affected:

Interference task: In the interference condition, the same stimuli as in the encoding task were presented again, but at a different position. Thus, the spatial contextual information was expected to interfere with that of the initial encoding task. Participants were asked to decide whether the object was natural or artificial but to ignore the spatial position.

Reminder task: In the reminder condition, the stimuli presented during encoding were presented again, albeit in the middle at the bottom of the screen. Thus, no interference with spatial contextual information was assumed. Instead, repeated presentation of the stimuli at a neutral position was expected to facilitate memory consolidation by causing reactivation of the memory for the respective position during the preceding encoding run. Participants were asked to make natural-artificial judgments. They were not instructed to recall the position of the stimuli from the encoding task.

Control task: In the control task, abstract and pixelated images with no meaningful information were presented in the middle at the bottom of the screen. Participants were asked to press a button with their right index finger each time an image appeared. The task was expected to provide neither interference nor any type of reminder, while variables such as motor activity and visual input were kept constant to the interference and reminder task.

For each of these modulatory tasks, the number of stimuli, duration of presentation, and inter-trial interval were identical to the encoding task.

During the retrieval task, the stimuli of the encoding task were presented in the screen’s center. Participants had to indicate the position of the stimuli during encoding by pressing four respective keys with their right-hand fingers. Each stimulus was presented for 2 s, followed by a variable inter-trial interval of 2 to 12 s and a 2-s fixation cross.

### Post-scan Questionnaires

At the end of each of the three experiments, participants were asked whether they had fallen asleep. All participants reported staying awake during the three resting-state scans of each experiment, except one participant who reported falling asleep during the R3 session of the reminder condition. Additionally, participants were asked whether or not they intentionally rehearsed the learned material during the resting-state sessions. All participants negated active rehearsal of the information.

### Magnetic Resonance Imaging Data Acquisition

Functional MR images were acquired using a Siemens TRIO 3-Tesla whole-body scanner (Siemens, Erlangen, Germany) with a 12-channel phase array head coil for enhanced overall signal and transmission properties. T2*-weighted echo-planar images (EPI) with BOLD contrast were obtained using the following sequence parameters: time of repetition (TR) = 2.43 s, echo time (TE) = 30 ms, number of slices = 40 axial slices, slice thickness = 3 mm, slice order = interleaved ascending, flip angle = 90°, field of view = 200 mm, matrix size = 64 × 64, in-plane resolution/pixel size = 3.1 mm × 3.1 mm. The rsfMRI scans lasted 7 min, acquiring 180 volumes. Additionally, a T1-weighted, anatomical image was obtained for each participant using a three-dimensional magnetization-prepared, rapid acquisition gradient echo (MP-RAGE) sequence (1 mm × 1 mm × 1 mm).

### Image Processing

To assess the physiological integrity of the brain and to check for structural changes indicating neurodegenerative diseases, the medial temporal lobe atrophy (MTA) score (Scheltens et al., 1992) and the Koedam score for atrophy of the parietal lobe (Koedam et al., 2011) were evaluated on T1-weighted anatomical images for each participant. Additionally, the Fazekas scale for white matter hyperintensities was assessed on T2-FLAIR images. No positive scores on the MTA scale were found. Two HS had a positive Koedam score, indicating a substantial widening of the sulci and a volume reduction of the gyri in the parietal lobe. In addition, two HS had a Fazekas score of two, indicating moderate aging-related changes in white matter and thus a higher risk of developing cognitive impairment (Inzitari et al., 2007). However, since no cognitive impairment was evident in these participants, they remained in the sample.

Preprocessing of structural data was performed using the Computational Anatomy Toolbox (CAT12, Version 12.1^1^), an extension of Statistical Parametric Mapping software (SPM12^2^). CAT12 was used with default settings, described in detail in the CAT12 manual^3^. Preprocessing steps included: (1) bias field inhomogeneity correction, (2) skull stripping, (3) segmentation, (4) spatial normalization to the MNI space using the Diffeomorphic Anatomical Registration Through Exponentiated Lie algebra (DARTEL) algorithm (Ashburner, 2007), and (5) resampling at an isotropic resolution of 1.5 mm.

Preprocessing of functional data was performed using CONN functional connectivity toolbox 18.a (Whitfield-Gabrieli and Nieto-Castanon, 2012). In short, preprocessing steps included: (1) slice time correction, (2) motion correction (realigning and unwarping), (3) outlier detection (scans with an absolute displacement relative to the previous volume greater than 0.9 mm were defined as an outlier), (4) co-registration to the structural data, (5) normalization by applying the deformation field maps of the structural data, and (6) spatial smoothing using an 8 mm full-width at half-maximum Gaussian kernel.

Subsequently, to improve sensitivity and reliability of the rsfMRI analyses, linear regression was used to remove the effects of nuisance variables, including realignment with six rigid-body parameters describing the motion of each participant (translation in the x-, y-, z-axis and rotation about the x-, y-, z-axis) and their first-order derivatives, and scrubbing with the images detected as outliers (see Outlier Detection). For the white matter signal and cerebrospinal fluid signal, a component-based noise correction method (CompCor) was applied to the data (Behzadi et al., 2007). Finally, bandpass filtering to 0.008–0.09 Hz and detrending were performed.

A comparison of the estimated distribution of correlation coefficients between randomly selected pairs of points in the brain before and after noise reduction confirmed that preprocessing and denoising successfully minimized the influence of artifactual factors on FC measurements (Supplementary Figure 1).

Mean motion as framewise displacement (Framewise Displacement as per Jenkinson et al., 2002) ranged from 0.11 to 0.13 mm (*SD* = 0.07–0.10) in each of the sessions. A 2 × 3 × 3 ANOVA with condition, session, and group as factors indicated a significant main effect session [*F(2,54)* = 10.77, *p* < 0.001]. *Post-hoc* analyses revealed significantly higher FD for session 2 and 3 compared to session 1 in all conditions (both *p* < 0.01). In addition, older individuals showed higher FD compared to younger individuals across all sessions and conditions [*F(1,27)* = 14.15, *p* < 0.001]. A summary of the individual means and standard deviations can be found in Supplementary Table 2.

### Resting-State Functional Magnetic Resonance Imaging Analysis

On the one hand, we aimed to investigate resting-state (RS) activity changes as a function of early memory consolidation processes without *a priori* assumptions. On the other hand, we were interested in how these areas of changing activity interact in networks during consolidation. Therefore, a two-step analysis was performed: First, the fractional amplitude of low-frequency fluctuations (fALFF) was calculated to analyze consolidation-related neural activity. Second, a region of interest (ROI)-to-ROI analysis was performed using the fALFF clusters from the previous analysis as seed ROIs.

The rsfMRI analyses were performed in CONN Connectivity Toolbox. Significant results are reported at an FDR-corrected cluster-level threshold of *p* < 0.05 (cluster forming threshold *p* < 0.001, one-tailed). Analyses across both groups were corrected for age. Education was not included as an additional covariate based on the high correlation between age and education (Supplementary Table 3). No correlation between gender and memory performance was found neither across groups nor within groups (both groups: *r* = 0.294, *p* = 0.121, *95% CI* [−0.121, 0.664]; HS: *r* = −2.72, *p* = 0.309, *95% CI* [−0.717, 0.284]; HY: *r* = 0.439, *p* = 133, *95% CI* [−0.173, 0.860]). Also, no gender effect was found with respect to resting-state activity, neither during post-encoding rest nor when compared to pre-encoding rest. For this reason, gender is also not included as a covariate of no interest. All analyses were corrected for the session-specific mean motion as assessed by mean FD Jenkinson (Jenkinson et al., 2002). As the behavioral results indicated that the modulating task during the consolidation phase of the reminder condition did not induce the expected effect on memory performance, rsfMRI analyses were performed only for the data of the control and interference condition, but not for the data of the reminder condition.

#### Fractional Amplitude of Low-Frequency Fluctuations Analysis

Unlike other voxel-wise measures, fALFF does not reflect FC but rather the amplitude of neural activity during the resting state. FC analysis can provide us with information about a set of brain regions within a network, but it does not provide information about the change in regional activity. Furthermore, abnormal connectivity between brain regions does not allow us to determine exactly in which brain region the spontaneous activity is abnormal. Therefore, the analysis of low-frequency fluctuations using fALFF allows us to predict changes in intrinsic spontaneous brain activity in specific brain regions in older compared to younger individuals during memory consolidation.

In order to observe spontaneous and intrinsic brain activity during early memory consolidation, we calculated fALFF for pre-encoding rest (R1), and immediate (R2), and delayed post-encoding rest (R3). fALFF was calculated as the ratio of the root mean square of the BOLD signal within the frequency band of interest (0.008–0.09 Hz) relative to the entire frequency spectrum. Compared to the amplitude of low-frequency fluctuations (ALFF), fALFF is considered to offer higher sensitivity and specificity for spontaneous brain activity and is less prone to physiological noise (Zou et al., 2008). To account for interindividual differences, the subject-level voxel-wise fALFF maps were transformed into z-score maps by subtracting the mean fALFF obtained for the entire brain and then dividing it by its standard deviation.

A comparison between fALFF in CON R1 and CON R2 as well as CON R1 and CON R3 using paired *t*-tests informs about consolidation-related activity. The additional comparison between CON R2 and CON R3 indicates how this consolidation-related activity changes with time. To examine the influence of the interference task on consolidation-related activity, fALFF was compared between INT R1 and INT R3 as well as INT R2 and INT R3 using paired *t*-tests. To analyze the effect of age on consolidation-related resting-state activity changes, we performed interaction analyses with the factors *group* and *session* for both control and interference conditions.

**FIGURE 2:**
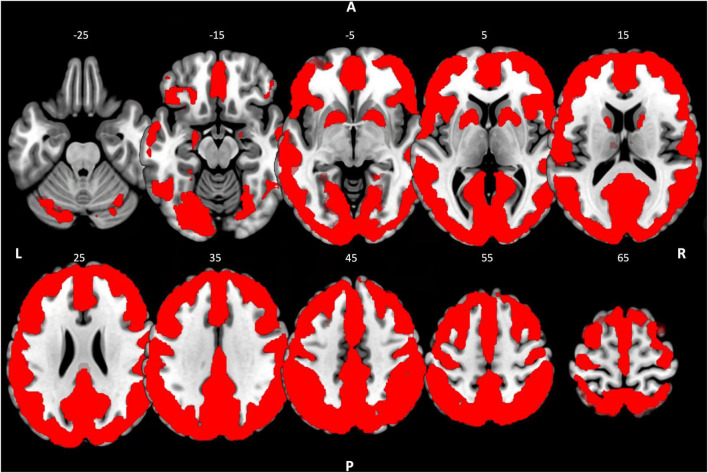
Resting-state activity map. Binarized resting state activity map resulting from a one-sample *t*-test across groups with the concatenated fALFF-maps of R1, R2, and R3 of the control condition. Cluster-level threshold pFDR < 0.05. R, right; L, left; A, anterior; P, posterior.

To ensure that the contrasts described above did not result in changes in activity in brain regions where no significant activity was originally observed in R1, R2, or R3, the contrasts were masked. The masks used were binarized activity maps of the resting-state sessions, which were included in the respective contrast. For example, the contrast between CON R1 and CON R2 was calculated in a mask, which was a binarized activity map of the resting state activity during CON R1 and CON R2. The activity map resulted from a one-sample *t*-test across sessions CON R1 and CON R2. Clusters that exceeded the cluster-level threshold (p-FDR < 0.05) formed the respective resting activity map (Figure 2). However, to check for possible bias due to this approach, we also performed the across-group contrasts at the whole-brain level. The results were consistent, except for the findings in the cerebellum (Supplementary Table 4).

Subsequently, the mean fALFF values from the clusters that differed significantly in resting-state activity between R1 and R2/R3 of the control condition were exported, corrected for motion, and further analyzed in SPSS (IBM SPSS Statistics; version 25, IBM Corp., Armonk, NY, United States). Using multiple regression analysis, we examined correlations with memory performance measures.

#### Region of Interest-to-Region of Interest Analysis

In addition to consolidation-related resting-state activity changes from pre- to post-encoding rest, we were interested in the functional dynamics between the identified consolidation-related brain regions and large-scale brain networks during the consolidation phase. Therefore, we conducted a subsequent ROI-to-ROI analysis. The results of the fALFF analysis regarding the contrasts between the sessions of the control condition indicate that the consolidation process progresses from immediate to delayed post-encoding rest. The most inclusive activity changes could be observed between pre-encoding rest and delayed post-encoding rest. Therefore, the significant clusters of this contrast were used as seeds in the following ROI-to-ROI analysis. A memory network (literature-based selection of regions) and the predefined network ROIs of the CONN toolbox, which were derived from ICA analyses of the HCP dataset (497 participants) related to cognitive functions, were used as targets:

– *MTL memory network* (Ward et al., 2014) — bilateral hippocampus and bilateral anterior and posterior parahippocampal cortex and bilateral anterior and posterior middle temporal gyrus.
– *Default mode network (DMN; CONN)* — medial prefrontal cortex, bilateral lateral parietal cortex, precuneus.
– *Salience network (SN; CONN)* — anterior cingulate cortex, bilateral anterior insula, bilateral rostral prefrontal cortex, bilateral supramarginal gyrus.
– *Visual network (VN; CONN)* — calcarine sulcus, occipital pole, bilateral lateral occipital gyrus.
– *Dorsal Attention network (DAN; CONN)* — bilateral frontal eye field, bilateral inferior parietal cortex.
– *Central Executive network (CEN; CONN)* — bilateral lateral prefrontal cortex, bilateral posterior parietal cortex.

Region of interest-to-ROI FC matrices (with Fisher-transformed Pearson correlation coefficients between ROI-BOLD time series averaged over all ROI voxels) were calculated for each participant. First, we examined the integration of brain regions showing consolidation-related changes in resting-state activity into large-scale brain networks during early memory consolidation. To determine which functional connections between the fALFF-based seeds and network ROIs are significant during consolidation, we computed a one-sample *t*-test for the ROI-to-ROI correlation coefficients of the R3 session of the control condition, including both groups. Second, we examined the effect of interference on FC by calculating a paired *t*-test between FC in the R3 sessions of the control and interference conditions. Next, we analyzed the effect of age on FC. Therefore, we calculated a two-sample *t*-test to compare FC in the R3 session of the control condition.

To determine whether the observed connectivity pattern predicted memory performance, we computed a regression analysis for FC in the R3 session and memory performance in the control condition. In addition, we used multiple regression analysis to examine whether FC during the R3 session of the interference condition was related to memory performance and inhibition capacity in the interference condition. Only those ROI-to-ROI associations that showed FC during the R3 session of their respective condition were included in the multiple regression analyses. To examine group differences in the predictive value of FC for performance measures, the regression analyses were also calculated within groups. Subsequently, the correlation coefficients of the two groups were statistically compared using Fisher z-transformation.

### Behavioral Data Analysis

All behavioral data analyses were performed using IBM SPSS Statistics (version 25, IBM Corp., Armonk, NY, United States). Spatial contextual memory performance was operationalized by the percentage of correctly retrieved object-location associations. To evaluate the effects of age and interference, a mixed-effects ANOVA with the between-subjects factor *group* (*HY vs. HS*) and the within-subject factor *condition* (*control*, *reminder*, and *interference condition*) was calculated.

We further analyzed the type of incorrect spatial contextual judgments in the interference condition. Two types of errors were classified: (1) intrusions, defined as trials in which the object-location association of the interference and not the object-location association of the encoding task was remembered, and (2) general errors, defined as trials in which judgments did not match the object position during either the interference or encoding task. First, a one-sample *t*-test was performed to evaluate whether the number of intrusions relative to the total number of incorrect responses was significantly different from chance and thus whether the experimental condition induced interference. Second, the total number of intrusions was examined for group differences using a two-sample *t*-test. Inhibition capacity or interference susceptibility was operationalized as d-prime:


d-prime=z(INTcorrectretrieval)-z(intrusions)

Positive d-prime values indicate the ability to successfully ignore the object-location associations from the interference task (inhibition capacity). A negative d-prime, in turn, indicates susceptibility to the object-location associations from the interference task (susceptibility to retroactive interference). A two-sample *t*-test analyzed group differences in d-prime.

Analyses including both groups were corrected for age. Education was not included as an additional covariate based on the high correlation between age and education. For an overview of the correlations of *age* and *education* with *behavioral performance* in all three experimental conditions, see Supplementary Table 3. Since no correlation was found between gender and memory performance either across or within groups (both groups: *r* = 0.294, *p* = 0.121, *95% CI* [−0.121, 0.664]; HS: *r* = −2.72, *p* = 0.309, *95% CI* [−0.717, 0.284]; HY: *r* = 0.439, *p* = 133, *95% CI* [−0.173, 0.860]), gender was also not included as an additional covariate. To avoid violations of the assumption of normality, we used bias-corrected and accelerated (BCa) bootstrapping (with 5000 iterations) whenever possible. However, bootstrapping is not implemented for repeated measures ANOVA in SPSS. Nevertheless, according to simulation studies, ANOVAs are robust against the violation of this assumption (Berkovits et al., 2000). *P*-values were corrected with the FDR method whenever necessary to account for multiple testing.

## Results

### Behavioral Data

**FIGURE 3:**
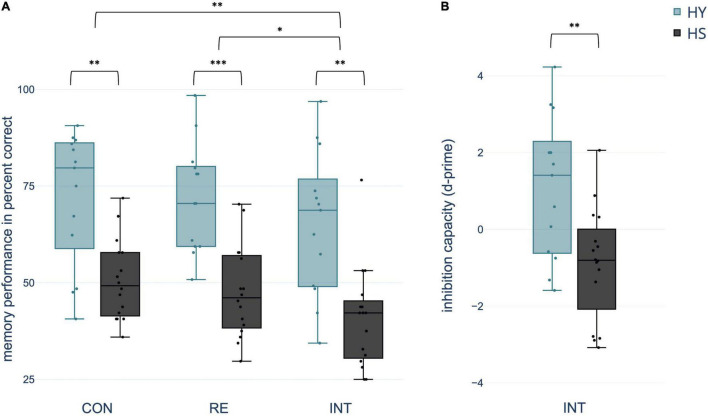
Differences between groups and conditions **(A)** regarding spatial context memory performance in percent correct and **(B)** inhibition capacity operationalized in *d*-prime [z(correct retrieval)–z(intrusions)]. CON, control condition; RE, reminder condition; INT, interference condition; HY, healthy young; HS, healthy seniors. *p < 0.05; ^**^p < 0.01; ^***^p < 0.001.

Mixed ANOVA yielded a significant within-subject effect *condition*. Memory performance varied with the extent of interference during consolidation across groups [*F*(2,54) = 6.901, *p* = 0.002, *η^2^* = 0.204]. Pairwise comparisons indicated a significantly decreased memory performance in the interference relative to both the control and reminder conditions (both *p-FDR* < 0.018). However, there was no significant difference in memory performance between the control and reminder conditions (*p-FDR* = 0.450). In addition, the effect *group* was significant with better memory performance in HY compared to HS across conditions [*F*(1,27) = 26.509, *p* < 0.0001, *η^2^* = 0.495] (Figure 3). ANOVA revealed no significant condition*group interaction (*p* > 0.05).

In the interference condition, 48.54% of the total number of mistakes were classified as intrusions. Considering that the chance probability for this kind of mistake among all possible false responses was 1/3, the percentage of intrusions was significantly above chance (test score: 33.33%, *t*(28) = 5.437, *p* < 0.001, *95% CI* [9.377, 20.403], *Cohen’s d* = 1.01, one-sample *t*-test). HS showed significantly more total intrusions than HY [*t*(27) = −2.193, *p* = 0.037, *95% CI*[−10.973, −1.231], *Cohen’s d* = −0.819, two-sample *t*-test].

Using *d*-prime, the number of intrusions was related to the number of correctly remembered object-location associations in the interference condition. A two-sample *t*-test showed a significant difference in d-prime between groups, indicating a negative d-prime for HS and a positive d-prime for HY [*t*(27) = 3.209, *p* = 0.007, *95% CI* [0.673, 3.359], *Cohen’s d* = 1.198] (Figure 3).

### Resting-State Functional Magnetic Resonance Imaging Data

#### Fractional Amplitude of Low-Frequency Fluctuations Analysis

The comparison of fALFF during the resting-state sessions of the control condition revealed an increase in resting-state activity from R1 to R3 within (1) the right lingual gyrus (LG), (2) left occipital pole (OP), (3) left LG, and (4) right OP and a decrease within the (1) left supramarginal gyrus (SMG), (2) right middle temporal gyrus (MTG), and (3) left precuneus (Pc). Further comparisons between R1 and R2 and R2 and R3 revealed overlapping but less significant results (Table 3). The comparison of fALFF during the resting-state sessions of the interference condition revealed a decrease in resting-state activity from R1 to R2 in the SMG, precuneus, superior frontal gyrus, and inferior frontal gyrus. A significant decrease in activity in the precuneus was also observed from R2 to R3. Comparing R1 to R3 only an increase in resting-state activity was observed in the right occipital pole (Table 4).

**TABLE 3:** Consolidation-specific resting-state activity.

region		x,y,z {mm}	Peak p-unc	*T*-value	cluster size	cluster p-FDR	Cohen’s *d*
**(A) CON R1 > R2**							
MTG	R	54 −58 −12	<0.001	5.71	37	0.018	0.981
**(B) CON R2 < R3**							
LG	L	−4 −76 10	<0.001	5.05	133	<0.001	1.073
**(B) CON R2 > R3**							
SMG	L	−54 −42 34	<0.001	4.6	18	0.051	0.845
temporal pole	R	58 18 −2	<0.002	4.56	14	0.051	0.943
superior frontal gyrus	R	2 56 2	<0.003	4.27	21	0.051	0.775
Precuneus	R	14 −48 14	<0.004	4.25	31	0.008	0.810
**(C) CON R1 < R3**							
LG	R	12 −66 2	<0.001	5.87	121	<0.001	1.101
occipital pole	L	−14 −92 6	<0.001	5.26	93	<0.001	1.224
LG	L	−2 −74 8	<0.001	4.88	78	<0.001	0.828
occipital pole	R	16 −92 0	<0.001	4.51	89	<0.001	0.901
**(D) CON R1 > R3**							
SMG	L	−50 −38 32	<0.001	6.72	80	0.001	1.224
MTG	R	58 −52 −8	<0.001	5.71	69	0.011	1.007
Precuneus	L/R	0 −60 30	<0.001	5.37	80	0.001	0.891

*Brain regions showing a significant increase or decrease in fALFF from pre-(R1) to immediate (R2) or delayed post-encoding rest (R3) in the control condition (CON), indicating consolidation-related activity in the resting state and providing information about the time course of early memory consolidation. Clusters are reported at pFDR < 0.05. L, left; R, right; SMG, supramarginal gyrus; MTG, middle temporal gyrus; LG, lingual gyrus.*

**TABLE 4:** Effect of interference on consolidation-related resting-state activity.

region		x,y,z {mm}	Peak p-unc	*T*-value	cluster size	cluster p-FDR	Cohen’s *d*
**(A) INT R1 > R2**							
inferior frontal gyrus	R	42 32 −6	<0.001	5.81	43	0.005	1.016
SMG	L	−42 −50 24	<0.001	4.92	28	0.026	0.838
Precuneus	L/R	0 −52 42	<0.001	4.67	34	0.005	0.804
superior frontal gyrus	R	4 54 0	<0.001	4.59	37	0.005	0.759
superior frontal gyrus	L	−4 52 10	<0.001	4.37	75	0.005	0.994
**(B) INT R2 > R3**							
Precuneus	R	6 −58 54	<0.001	5.89	114	0.001	1.089
Precuneus	R	10 −68 36	<0.001	5.04	50	0.001	0.977
**(C) INT R1 < R3**							
occipital pole	R	12 −86 18	<0.001	7.4	68	0.019	0.948

*Brain regions showing a significant increase or decrease in fALFF from pre- (R1) to immediate (R2) or delayed post-encoding rest (R3) in the interference condition (INT). Activity changes from R1 to R2 indicating consolidation-related activity in the resting state and activity changes from R2 to R3 providing information about the effect of the interference task on consolidation processes. Clusters are reported at pFDR < 0.05. L, left; R, right; SMG, supramarginal gyrus.*

Interaction analyses for the control condition showed no significant results. However, at a lower threshold, group differences in the interaction became apparent, covering regions for which we also observed changes in resting-state activity across groups (Table 5). The interaction analyses for the interference condition showed a significantly higher decrease in activity from R2 to R3 in the left SMG and left middle frontal gyrus (MFG) for HY compared to HS (Table 6).

**TABLE 5:** Interaction analyses with factors session and group for CON RS activity.

region		x,y,z {mm}	Peak p-unc	*T*-value	cluster size	Cluster p-FDR	Cohen’s *d*
**(A) HY > HS – CON R1 > R2**							
OP	R	12 −84 12	<0.001	4.6	29	0.172	1.512
**(B) HY > HS – CON R1 > R3**							
MTG	L	−66 −24 −6	<0.001	5.24	22	0.131	0.870

*Results of 2 × 2 ANOVAs with the factors session (R1 vs. R2; R2 vs. R3; R1 vs. R3) and group (HY vs. HS) for RS activity of the control condition (CON). Brain regions showing different activity changes from session to session between groups. Clusters are reported at p-unc. < 0.001. RS, resting-state; R1, pre-encoding rest; R2, immediate post-encoding rest; R3, delayed post-encoding rest; L, left; R, right; OP, occipital pole; MTG, middle temporal gyrus.*

**TABLE 6:** Interaction analyses with factors session and group for INT RS activity.

region		x,y,z {mm}	Peak p-unc	*T*-value	cluster size	Cluster p-FDR	Cohen’s *d*
**(A) HY > HS – INT R2 > R3**							
MFG	L	−48 16 36	<0.001	5.32	52	0.026	1.280
SMG	L	−62 −42 34	<0.001	5.7	38	0.026	1.147

*Results of 2 × 2 ANOVAs with the factors session (R1 vs. R2; R2 vs. R3; R1 vs. R3) and group (HY vs. HS) for RS activity of the interference condition (INT). Brain regions show different activity changes from session to session between groups. Clusters are reported at p-FDR < 0.05. RS, resting-state; R1, pre-encoding rest; R2, immediate post-encoding rest; R3, delayed post-encoding rest; L, left; R, right; MFG, middle frontal gyrus; SMG, supramarginal gyrus.*

**FIGURE 4:**
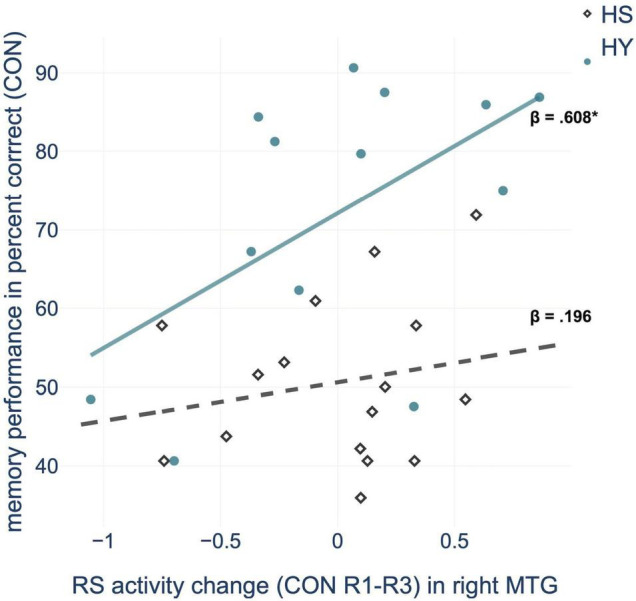
Regression analysis showed that consolidation-related resting-state (RS) activity changes in the middle temporal gyrus (MTG) from pre-(R1) to post-encoding rest (R3) predict memory performance in the control condition (CON), for HY (healthy young) but not for HS (healthy seniors). *p < 0.05.

Regression analysis showed that consolidation-related resting-state activity changes within the right MTG significantly predicted memory performance in the control condition. Further within-group analyses confirmed this relationship for HY but not for HS (Figure 4 and Supplementary Table 5). For HY, a greater decrease in resting-state activity from CON R1 to CON R3 was associated with better memory performance. Resting-state activity changes in the other consolidation-related brain regions did not significantly predict memory performance. For the interference condition, no resting-state activity changes within consolidation-related brain regions could predict memory performance or inhibition capacity (all *p* > 0.05).

#### Region of Interest-to-Region of Interest Analysis

**FIGURE 5:**
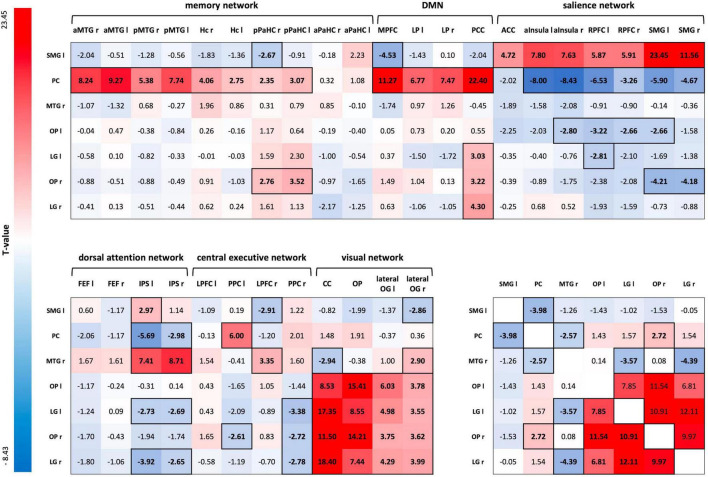
ROI-to-ROI connectivity matrix for the R3 session of the control condition. *T*-values of one-sample *t*-tests of all ROI-to-ROI associations for delayed post-encoding rest (R3) of the control condition. Significant *T*-values are bold and outlined (*p-FDR* < 0.05). Cells are colored red for positive coupling and blue for negative coupling between regions showing consolidation-related changes in resting-state activity (seed ROIs) and nodes of large-scale brain networks (target ROIs). SMG, supramarginal gyrus; PC, precuneus; MTG, middle temporal gyrus; OP, occipital pole; LG, lingual gyrus; aMTG, anterior MTG; pMTG, posterior MTG; Hc, hippocampus; pPaHC, posterior parahippocampal cortex; aPaHC, anterior parahippocampal cortex; MPFC, medial prefrontal cortex; LP, lateral parietal cortex; PCC, posterior cingulate cortex (including the Pc); ACC, anterior cingulate cortex; aInsula, anterior insula; RPFC, rostral prefrontal cortex; FEF, frontal eye field; IPS, inferior parietal cortex; LPFC, lateral prefrontal cortex; PPC, posterior parietal cortex; CC, calcarine cortex; lateral OG, lateral occipital gyrus; ROI, region of interest.

A one-sample *t*-test for the ROI-to-ROI correlation coefficients of the R3 session of the control condition, including both groups, showed positive and negative coupling of consolidation-related regions with nodes of large-scale brain networks during early memory consolidation. Of particular importance was the integration of the SMG-seed into the salience network (SN) and the anticorrelation to parts of the default mode network (DMN). On the other hand, the Pc-seed was strongly connected to nodes of the memory network and the DMN, and showed an anticorrelation to large parts of the SN. In addition, there was strong connectivity of OP and LG of both hemispheres with each other and with nodes of the visual network. The left LG, right OP, and right LG also positively correlated with the posterior cingulate cortex (PCC) as a core region of the DMN (Figure 5; for FDR-corrected *p*-values, see Supplementary Figure 2).

A two-sample *t*-test for CON R3 revealed a significantly different FC between the SMG-seed and a node of the memory network – the right anterior MTG – for HY and HS. HY showed positive coupling between the SMG-seed and the right anterior MTG, while HS demonstrated negative coupling [*M*_*HY*_ = 0.071, *SD*_*HY*_ = 0.132; *M*_*HS*_ = −0.058, *SD*_*HS*_ = 0.132; *t*(26) = 3.40, *p-FDR* = 0.0446, *Cohen’s d* = 1.125]. Applying a more liberal threshold, there were also group differences in FC between the SMG seed and additional nodes of the memory network - the left anterior MTG [*t*(26) = 2.03, *p-unc* = 0.026, *Cohen’s d* = 0.378] and left posterior MTG [*t*(26) = 2.11, *p-unc* = 0.022, *Cohen’s d* = 0.295].

**FIGURE 6:**
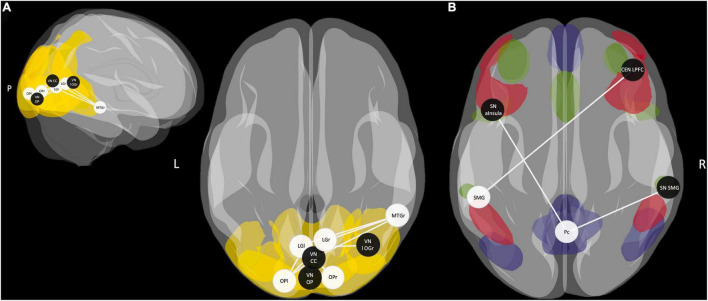
**(A)** Functional connectivity (FC) between occipital, inferior temporal, and middle temporal regions during post-encoding rest predicted memory performance in the control condition (*p*-FDR < 0.05). Applying a more liberal threshold (*p* < 0.05), this network extends to additional nodes within occipital and temporal regions. Areas of the visual network (VN) are highlighted in yellow. **(B)** FC between the SMG seed as part of the salience network (SN) and a node of the central executive network (CEN) and connections between the Pc seed as a core area of the default mode network (DMN) and nodes of the SN predicted memory performance in the control condition (*p*-FDR < 0.05). Areas of the DMN are highlighted in blue, areas of the CEN in red, and areas of the SN in green. fALFF-derived seeds are represented by white spheres and network ROIs by black spheres. SMG, supramarginal gyrus; Pc, precuneus; MTG, middle temporal gyrus; OP, occipital pole; LG, lingual gyrus; aInsula, anterior Insula; LPFC, lateral prefrontal cortex; CC, calcarine sulcus; l OG, lateral occipital gyrus; r, right; l, left; P, posterior.

Multiple regression analysis revealed a significant predictive value of FC between occipital, inferior temporal, and medial temporal regions for memory performance in the control condition (*p*-FDR < 0.05). However, the correlations of ROI-to-ROI associations with memory performance did not survive FDR correction across seeds. Applying a more liberal threshold (*p* < 0.05), this network extended to additional nodes within occipital and temporal regions. Besides, FC between the SMG-seed and a node of the central-executive network (CEN) – right lateral prefrontal cortex (LPFC) – and FC between the Pc-seed and nodes of the SN – right SMG and left anterior insula – showed to be predictive of memory performance in the control condition (Figure 6). Overall, almost all ROI-to-ROI associations showed, at least descriptively, a higher predictive value for memory performance in the HY than in the HS group (Table 7).

**TABLE 7:** Functional connectivity during post-encoding rest predicting memory performance of HY and HS in the control condition.

		HY+HS				HY		HS		Compare β	
Seed-ROI	Target-ROI	Statistic (CONN)	β	*p*-value	p-FDR	β	*p*-value	β	*p*-value	z-Diff	*p*-value
MTG	VN – calcarine sulcus	T(25) = 2.18	0.324	0.019*	0.047*	0.498	0.052	0.247	0.205	0.700	0.121
MTG	LG r	T(25) = 2.45	0.357	0.011*	0.047*	0.558	0.032*	0.194	0.250	1.030	0.076
MTG	LG l	T(25) = 2.16	0.339	0.020*	0.047*	0.410	0.093	0.309	0.184	0.276	0.196
OP l	VN – calcarine sulcus	T(25) = 3.34	0.443	0.001***	0.009*	0.853	0.000***	0.284	0.155	2.318	0.005**
OP l	LG r	T(25) = 2.47	0.359	0.010**	0.036*	0.661	0.010**	0.341	0.118	1.045	0.074
OP l	LG l	T(25) = 1.93	0.292	0.032*	0.075	0.551	0.032*	0.374	0.090	0.539	0.148
LG l	VN – occipital pole	T(25) = 1.86	0.272	0.037*	0.121	0.486	0.060	0.288	0.150	0.557	0.145
LG l	VN – lateral OG r	T(25) = 2.71	0.423	0.006**	0.079	0.436	0.078	0.709	0.007**	−0.994	0.080
LG l	OP r	T(25) = 1.71	0.269	0.049*	0.129	0.498	0.052	0.203	0.235	0.810	0.105
OP r	VN – calcarine sulcus	T(25) = 2.05	0.311	0.026*	0.321	0.711	0.005**	0.145	0.304	1.767	0.020*
LG r	VN – occipital pole	T(25) = 2.12	0.303	0.022*	0.089	0.539	0.038*	0.271	0.165	0.772	0.110
SMG	CEN – LPFC r	T(25) = −2.5	−0.399	0.010**	0.122	−0.89	0.001***	0.356	0.150	2.542	0.003**
Pc	SN – SMG r	T(25) = 1.99	0.291	0.029*	0.417	0.429	0.085	0.072	0.404	0.919	0.090
Pc	SN – aInsula l	T(25) = 1.85	0.276	0.038*	0.417	0.327	0.154	0.327	0.132	0.000	0.250

Multiple regression analysis showed a significant predictive value of ROI-to-ROI connectivity for memory performance in the control condition. Overall, almost all ROI-to-ROI associations showed, at least descriptively, a higher predictive value for memory performance of healthy young individuals (HY) than for memory performance of healthy seniors (HS). SMG, supramarginal gyrus; Pc, precuneus; MTG, middle temporal gyrus; OP, occipital pole; LG, lingual gyrus; VN, visual network; SN, salience network; aInsula, anterior Insula; CEN, central executive network; LPFC, lateral prefrontal cortex; r, right; l, left. *p < 0.05; **p < 0.01; ***p < 0.001.

Regarding the interference effect, a paired *t*-test showed a significantly reduced negative coupling between the Pc-seed and a node of the SN – namely the left SMG [*t*(25) = −3.38, *p-unc* = 0.001, *p-FDR* = 0.047, *Cohen’s d* = −0.655] – and marginally significantly reduced positive coupling between the Pc-seed and left and right OP [OP l: *t*(25) = 3.04, *p-unc* = 0.003, *p-FDR* = 0.053, *Cohen’s d* = 0.6140; OP r: *t*(25) = 3.17, *p-unc* = 0.002, *p-FDR* = 0.053, *Cohen’s d* = 0.6414] during INT R3 compared to CON R3. An interaction analysis revealed that the effect of interference on FC was irrespective of age.

**FIGURE 7:**
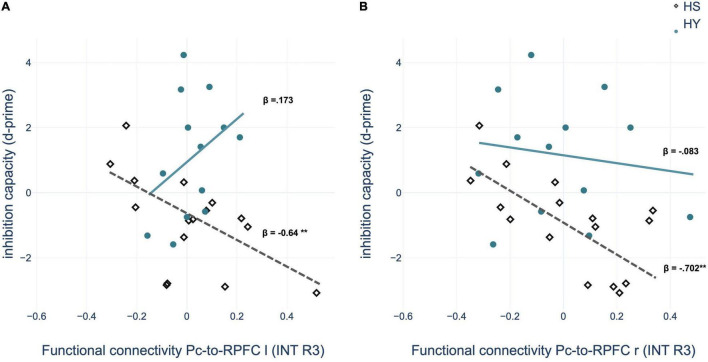
Regression analysis showed that inhibition capacity is predicted by region of interest (ROI)-to-ROI connectivity between left precuneus (Pc) and **(A)** left rostral prefrontal cortex (RPFC) and **(B)** right RPFC during post-encoding rest in the interference condition for HS (healthy seniors) but not for HY (healthy young). INT, interference condition. *p < 0.05; **p < 0.01; ***p < 0.001.

Multiple regression analyses within groups showed a significant predictive value of the FC between the Pc seed and nodes of the SN – bilateral rostral prefrontal cortex (RPFC) – for memory performance after interference and interference susceptibility of HS. Thus, negative coupling predicted better memory performance and lower interference susceptibility (Table 8 and Figure 7). However, the correlations of the ROI-to-ROI associations with memory performance and interference susceptibility did not survive FDR correction across seeds. We observed no correlation between FC and behavioral measures of the interference condition for HY.

**TABLE 8:** Functional connectivity during post-encoding rest predicting memory performance and *d*-prime of HS in the interference condition.

		HS				HY	
Seed-ROI	Target-ROI	Statistic (CONN)	β	*p*-value	p-FDR	β	*p*-value
**INT memory performance**							
Pc	SN – RPFC r	T(13) = −3.40	−0.748	0.001***	0.012*	−0.58	0.425
Pc	SN – RPFC l	T(13) = −2.85	−0.741	0.001***	0.012*	0.118	0.381
**INT *d*-prime**							
Pc	SN – RPFC r	T(13) = −3.40	−0.702	0.002**	0.043*	−0.083	0.394
Pc	SN – RPFC l	T(13) = −2.85	−0.64	0.007**	0.062	0.173	0.327

Multiple regression analyses within groups showed a significant predictive value of ROI-to-ROI connectivity after interference for memory performance and interference susceptibility of healthy seniors (HS) but not for healthy young individuals (HY). Pc, precuneus; SN, salience network; RPFC, rostral prefrontal cortex; r, right; l, left; *p < 0.05; **p < 0.01; ***p < 0.001.

## Discussion

### Consolidation-Related Processes Become Less Efficient With Age

We observed changes in resting-state activity between pre-encoding rest and delayed post-encoding rest within parietal, temporal and occipital regions. The contrasts between pre-encoding and immediate post-encoding rest as well as immediate and delayed post-encoding rest revealed overlapping but less significant results. Thus, we conclude that resting-state activity changes successively with the progression of early memory consolidation.

The brain regions defined as consolidation-related on the basis of our results are consistent with findings of previous studies showing that these regions are of central importance for episodic memory (Otten and Rugg, 2001; Laird et al., 2009; Cabeza et al., 2011; Kukolja et al., 2016; Schott et al., 2019). Wang et al. (2008) found similar brain regions to be correlated with spontaneous brain activity in the primary visual area (PVA) at rest. FC was observed for the occipital poles with the visual association areas (including LG), the Pc, the inferior/middle temporal gyrus, and the parahippocampal gyrus. Based on the functional attribution of these regions, Wang and colleagues suggested that PVA-related spontaneous activity could be attributed to memory-related mental imagery or visual consolidation. Because in our study resting-state activity was observed at rest and in contrast between pre- and post-encoding rest, our results provide further evidence for this hypothesis. We conclude that the coactivation of regions within a parieto-occipito-temporal network is central for consolidation processes, both in younger and older adults.

In the right MTG, resting-state activity changes predicted memory performance, but only in younger individuals. Therefore, it can be assumed that changes in resting-state activity within a parieto-occipito-temporal network, as a neurophysiological mechanism underlying consolidation, becomes at least partially less efficient with age and thus less predictive of episodic memory performance.

Subsequent FC analyses revealed comparable integration of consolidation-related brain regions (MTG, Pc, SMG, bilateral LG, and OP) into large-scale brain networks for younger and older individuals. We observed integration of the SMG-seed into the SN and anticorrelation to parts of the DMN. On the other hand, the Pc-seed was firmly integrated into the memory network and the DMN and negatively correlated to large parts of the SN. In addition, bilateral LG and OP seeds were highly intercorrelated and correlated with the visual network. We also observed FC of the right MTG seed with occipital and inferior-temporal regions, forming a visual ventral stream network, for which we observed a positive correlation with memory performance. The association was stronger in younger than older individuals, suggesting a decrease in the efficiency of this network for consolidation processes with age, subsequently leading to aging-related memory decline.

The two-stream hypothesis states that two distinct systems process visual sensory input. The dorsal stream is associated with processing the spatial location of the object relative to the perceiver. In contrast, the ventral visual stream, which leads from the primary visual area to the temporal lobe, is associated with the processing and recognition of objects (Ungerleider and Haxby, 1994). Recent research found stimulus-specific reactivation during post-encoding rest in visual areas that propagated along the ventral stream and predicted performance in a subsequent memory task (Deuker et al., 2013). Interestingly, Jacobs et al. (2015) found an age-related decrease in FC between the middle temporal gyrus and the ventral visual stream but failed to observe a correlation with behavioral performance. Thus, our results complement previous research, as we observed a correlation with memory performance for the FC of the MTG with bilateral lingual gyrus and calcarine sulcus. More importantly, our results extend those previous studies by showing further FC between the bilateral occipital pole, bilateral lingual gyrus, right lateral occipital gyrus, and calcarine sulcus as predictive for memory performance, with a more robust memory prediction for younger compared to older individuals.

It could be assumed that FC within the visual ventral stream network represents the neurophysiological correlate of ongoing mental imagery processes of the material to be subsequently retrieved or visual working memory. However, since even in the control condition the consolidation phase was interrupted by a sensory task unrelated to the material to be consolidated, the data suggest that the neural activity and connectivity during the post-encoding rest does not merely represent working memory processes but is attributable to memory consolidation.

Furthermore, our results show that the FC between the salience, central executive, and default mode networks can predict memory performance. Better memory performance was associated with lower negative FC between the Pc, a core region of the DMN, and parts of the SN. In contrast, higher negative FC between the SMG, as a core region of the SN, and parts of the CEN, was associated with better memory performance. On the other hand, Jacobs et al. (2015) found age-related dynamic reorganization exclusively between the DMN and the executive network during post-encoding rest, but no association of these dynamics with the salience network. However, previous research suggests that the SN serves as a switch between the DMN and CEN (Sridharan et al., 2008). In contrast to the DMN, which is primarily active at rest (Raichle et al., 2001), the CEN shows activation during complex cognitive tasks and high wakefulness (Seeley et al., 2007). Consistent with the triple network model of Menon (2011), our results suggest that an SN-mediated switch between DMN and CEN occurs during consolidation. As in our study the relationship between the three networks allowed for better prediction of memory performance in younger compared to older adults, we hypothesize that this consolidation-induced shift in the competition between large brain networks becomes less efficient with age.

We suggest that with increasing age, the neural mechanisms underlying consolidation do not decrease in quantity but quality concerning the effectiveness of the neural mechanisms for successful consolidation. Thus, we conclude that with increasing age, the effectiveness of resting-state activity within the MTG, FC between regions in the visual ventral stream, and FC between SN, DMN, and CEN decrease, contributing to aging-related memory decline.

### Between-Network Connectivity Predicts Aging-Related Increase in Interference Susceptibility

Our results for the interference condition showed a decrease in activity from pre-encoding to immediate post-encoding rest before the interference task within frontal regions, the SMG and Pc. This decrease in activity is consistent with the results from the control condition. After the interference task, however, divergent results emerge. In particular, there is no significant change in resting-state activity within the SMG, MTG, and LG. However, the successive decrease in activity within the precuneus is consistent with our results from the control condition. These findings suggest that the interference task suppresses some but not all of the neural mechanisms underlying consolidation.

Furthermore, our results showed reduced negative coupling between the Pc as a core region of the DMN and parts of the SN and reduced positive coupling between the Pc and the PVA/OP after interference for both younger and older adults. Accordingly, while interference does not affect the resting-state activity within the Pc, its integration into functional networks seems to be disturbed by interference.

This assumption is further supported by the results of the correlation analyses with memory performance and interference susceptibility. We observed a predictive value of the FC between the Pc, as a core region of the DMN, and bilateral RPFC, as parts of the SN, in older but not in younger individuals. Accordingly, our results provide a behavioral correlate for the neural mechanisms of consolidation modulated by interference. Since our behavioral analyses showed that younger individuals could suppress the interfering position in most cases, whereas older individuals were unable to do so, it could be speculated that due to compensatory neural mechanisms relevant to inhibition capacity, memory performance in younger individuals cannot be predicted by the FC of the Pc.

Overall, our results provide evidence that interference disrupts the neuronal processes of memory consolidation leading to impaired retrieval performance. Although the activity changes during the consolidation phase within the Pc persist after interference, our results nevertheless show that the FC of the precuneus with the salience network changes due to interference. In particular, the link to the salience network seems to be of great relevance for inhibition capacity, as it shows a predictive value for the memory performance of the older individuals, which is particularly weakened by interference.

### Repeated Reactivation Might Cause Reconsolidation

Repeated reactivation of activity patterns from encoding is thought to be a central consolidation mechanism (Squire and Alvarez, 1995). In the current study, repeated presentation of stimuli at a neutral position during consolidation was meant to serve as a cued reminder of the original position during encoding, leading to repeated reactivation and thus better memory performance. However, this hypothesis could not be confirmed, as memory performance after the cued reminders was not better but worse. The concept of reconsolidation provides a possible explanation. Memories are reactivated upon retrieval or by a reminder and thus become unstable and prone to interference before a reconsolidation process re-establishes stability (Nader and Hardt, 2009). Previous research suggests that memories explicitly brought to consciousness, as in the present study, induce reconsolidation (Walker et al., 2003).

We hypothesize that the cued reminders presentation in the current study led to the reactivation of the memory trace already formed during the post-encoding rest period before the intervention (R2). Therefore, the memory traces became unstable again and had to be re-consolidated. However, when the cued reminder was presented implicitly, and thus reactivation occurred spontaneously, it could provide a central mechanism for memory consolidation and strengthen the stabilization of the memory trace, as several studies showed (Diekelmann et al., 2011; Deuker et al., 2013).

### Strengths and Limitations

This study is the first to investigate the effect of gradually manipulated interference on consolidation processes in aging. In three conditions, the extent to which interference challenged consolidation was varied. To ensure a standardized protocol across the three conditions, post-encoding rest had to be interrupted by a distracting task in each condition, which challenged consolidation processes even in the control condition. However, the stimuli of the distracting task in the control condition were scrambled pictures unrelated to the learned material. Therefore, retroactive interference with the stimuli or their position from the encoding task in the control condition is highly unlikely. Moreover, this task ensured that the neural mechanisms involved during the post-encoding rest could be attributed to consolidation rather than working memory processes. Additionally, a resting period without sensory input after learning is scarce in everyday life. Therefore, the control condition in the present study might more adequately reflect conditions outside the experimental environment than a period of wakeful rest without any sensory input.

In the present study, interference concerned the spatial context of the objects initially presented in the encoding task. Therefore, memory performance was operationalized as the number of correctly recalled object positions. On the one hand, this is consistent with previous studies showing that spatial context memory is particularly sensitive to aging effects (Spencer and Raz, 1995; Kukolja et al., 2009). On the other hand, memory performance served as a behavioral correlate for successful consolidation. However, it can be assumed that the spatial context and the objects themselves were encoded and consolidated. Therefore, memory performance for the spatial context does not seem to be an entirely sufficient indicator of successful consolidation. Future studies should consider object memory in addition to spatial context memory.

Furthermore, performance in a delayed retrieval task does not represent an entirely sufficient indicator of successful memory consolidation since it does not allow differentiation between successful encoding and consolidation. Concerning the memory paradigm in the current study, there may be three reasons why the position of the stimuli in the memory task was not remembered correctly: (1) The information was not encoded in the first place, i.e., during the learning phase this information was not consciously perceived, which is why it was not transferred to short-term memory; (2) the information was forgotten because it was not or not sufficiently consolidated and thus did not enter long-term memory; and (3) the information was overwritten (3a) either already during the learning phase by subsequent learning content or (3b) during the intervention task in the consolidation phase (retroactive interference). Future studies should additionally include an immediate retrieval task before consolidation. First, the performance of an immediate retrieval task could provide information about successful encoding. Second, a difference score between performance on an immediate and a delayed recall task would provide greater specificity as an indicator of successful memory consolidation and could provide insight into how much of the learning material, after it has been successfully encoded, is forgotten later in the process.

Memory consolidation can take days, whereas in the present study, the consolidation phase had to be terminated after 14 min for temporal economic reasons. However, our data contribute to a growing body of literature by showing that system-level consolidation, i.e., dynamics between large-scale brain networks, occurs immediately after encoding and is related to successful consolidation in terms of better memory performance. To the best of our knowledge, this is the first study to investigate, without *a priori* assumptions, resting-state activity and, based on this, connectivity between large-scale brain networks. Since memory consolidation processes are difficult to assess as they are not associated with any task but occur spontaneously during wakeful rest or sleep, we consider our methodological approach valuable to the field.

Finally, the sample size is small and the results must be interpreted cautiously. In terms of reliable statistical effects, this study follows a within-subjects design, which reduces the number of subjects needed. In addition, potential confounding variables were thoroughly controlled, and conclusions were drawn based on robust results. Additionally, it is important to point out a methodological limitation regarding spatial smoothing. Although a larger kernel size is recommended for smaller samples, it can also lead to a shift in the local maxima. For our chosen kernel size of 8mm, an average shift of the local maxima of up to 5mm can be expected compared to a kernel size of 6mm. This fact must be taken into account when considering the reported results (Mikl et al., 2008).

## Conclusion

Our findings indicate that deficits in episodic memory are not only due to altered neural processes during encoding and retrieval but at least partly due to impaired consolidation. Our results demonstrate that FC within a visual ventral stream network and between SN, DMN, and CEN underlying early consolidation becomes increasingly inefficient with age. Furthermore, our results indicate an accelerated susceptibility for retroactive interference in older individuals with specific dynamics between the SN and DMN as a neurophysiological correlate. The present findings serve as a basis for future studies investigating reconsolidation processes and possible compensatory mechanisms regarding inefficient consolidation and increased interference susceptibility.

## Data Availability Statement

The raw data supporting the conclusions of this article are provided by the authors without reservation. The unthresholded t-maps of the fALFF contrasts can also be accessed at the following URL: https://identifiers.org/neurovault.collection:12346.

## Ethics Statement

The studies involving human participants were reviewed and approved by Ethics Commission of Cologne University’s Faculty of Medicine, Cologne, Germany. The patients/participants provided their written informed consent to participate in this study.

## Author Contributions

OO and JK designed the experiment. OR collected the data. RF analyzed the data and wrote the manuscript. JD, NR, HG, QB, GF, JK, and OO revised and approved the manuscript. All authors fully qualify for authorship and contributed significantly to the work.

## Conflict of Interest

The authors declare that the research was conducted in the absence of any commercial or financial relationships that could be construed as a potential conflict of interest.

## Publisher’s Note

All claims expressed in this article are solely those of the authors and do not necessarily represent those of their affiliated organizations, or those of the publisher, the editors and the reviewers. Any product that may be evaluated in this article, or claim that may be made by its manufacturer, is not guaranteed or endorsed by the publisher.

## Footnotes

1
http://dbm.neuro.uni-jena.de/cat/

2
https://www.fil.ion.ucl.ac.uk/spm/software/spm12

3
http://www.neuro.uni-jena.de/cat12/CAT12-Manual.pdf
