# Supplementary material for: Decreased Efficiency of Between-Network Dynamics During Early Memory Consolidation With Aging
Source: Front Aging Neurosci. 2022 May 16;14:780630. doi: 10.3389/fnagi.2022.780630 (PMC9148994; doi:10.3389/fnagi.2022.780630)
Supplement: Supplementary file 1 [file Table_1.docx]

Supplementary Material

**Supplementary Table 1.** Frequency distribution of randomized order of conditions.

|  | frequency | | |
| --- | --- | --- | --- |
| order of conditions | HY | HS | total |
| INT-RE-CON | 2 | 2 | 4 |
| INT-CON-RE | 2 | 3 | 5 |
| RE-INT-CON | 2 | 3 | 5 |
| RE-CON-INT | 3 | 2 | 5 |
| CON-INT-RE | 2 | 4 | 6 |
| CON-RE-INT | 2 | 2 | 4 |
| N total | 13 | 16 | 2 |

Abbreviation: HY, healthy young group; HS, healthy senior group; CON, control condition; RE, reminder condition; INT, interference condition.

**Supplementary Table 2. Framewise Displacement as per Jenkinson**

|  | group | Mean | SD |
| --- | --- | --- | --- |
| CON R1 | HY | 0.0605 | 0.02184 |
|  | HS | 0.1473 | 0.06761 |
|  | Total | 0.1084 | 0.06771 |
| CON R2 | HY | 0.0703 | 0.02169 |
|  | HS | 0.1666 | 0.08565 |
|  | Total | 0.1234 | 0.08067 |
| CON R3 | HY | 0.0745 | 0.02307 |
|  | HS | 0.175 | 0.09395 |
|  | Total | 0.1299 | 0.08687 |
| RE R1 | HY | 0.061 | 0.02629 |
|  | HS | 0.1621 | 0.09098 |
|  | Total | 0.1168 | 0.08572 |
| RE R2 | HY | 0.0822 | 0.03589 |
|  | HS | 0.1833 | 0.11693 |
|  | Total | 0.1379 | 0.10244 |
| RE R2 | HY | 0.0839 | 0.02853 |
|  | HS | 0.1842 | 0.11263 |
|  | Total | 0.1392 | 0.09861 |
| INT R1 | HY | 0.0694 | 0.02424 |
|  | HS | 0.1517 | 0.0925 |
|  | Total | 0.1148 | 0.08105 |
| INT R2 | HY | 0.0739 | 0.02844 |
|  | HS | 0.179 | 0.10695 |
|  | Total | 0.1319 | 0.09647 |
| INT R3 | HY | 0.0806 | 0.02953 |
|  | HS | 0.186 | 0.10986 |
|  | Total | 0.1387 | 0.0984 |

Abbreviation: HY, healthy young group; HS, healthy senior group; CON, control condition; RE, reminder condition; INT, interference condition; R1, pre-encoding rest, R2, immediate post-encoding rest; R3, delayed post-encoding rest.

**Supplementary Table 3.** Correlations between behavioral performance, age, and education

|  |  |  | age | education | CON performance | RE performance | INT performance | d-prime |
| --- | --- | --- | --- | --- | --- | --- | --- | --- |
| age | Pearson Correlation |  | 1 | -.436* | -.628** | -.726** | -.680** | -.579** |
|  | Sig. (2-tailed) |  |  | 0.018 | 0 | 0 | 0 | 0.001 |
|  | N |  | 29 | 29 | 29 | 29 | 29 | 29 |
|  | BCa 95% Confidence Interval | Lower | . | -0.676 | -0.844 | -0.861 | -0.833 | -0.758 |
|  |  | Upper | . | -0.165 | -0.363 | -0.544 | -0.498 | -0.352 |
| education | Pearson Correlation |  | -.436* | 1 | 0.343 | 0.309 | .476** | .473** |
|  | Sig. (2-tailed) |  | 0.018 |  | 0.069 | 0.103 | 0.009 | 0.009 |
|  | N |  | 29 | 29 | 29 | 29 | 29 | 29 |
|  | BCa 95 % Confidence Interval | Lower | -0.676 | . | 0.072 | -0.002 | 0.17 | 0.208 |
|  |  | Upper | -0.165 | . | 0.61 | 0.585 | 0.729 | 0.697 |

Note: * p < .05; ** p < .01; *** p < .001.

**Supplementary Table 4.** Consolidation-specific resting state activity (whole brain analysis).

| region |  | x,y,z {mm} | Peak p-unc | T | cluster size | Cluster p-FDR |
| --- | --- | --- | --- | --- | --- | --- |
| (A) CON R1 < R3 |  |  |  |  |  |  |
| LG | R | 14 -66 4 | <0.001 | 6.97 | 180 | <0.001 |
| occipital pole | L | -14 -92 6 | <0.001 | 5.26 | 100 | <0.001 |
| LG | L | -2 -70 4 | <0.001 | 4.99 | 93 | <0.001 |
| occipital pole | R | 16 -92 0 | <0.001 | 4.51 | 91 | <0.001 |
| (B) CON R1 > R3 |  |  |  |  |  |  |
| SMG | L | -50 -38 32 | <0.001 | 6.72 | 97 | 0.001 |
| MTG | R | 58 -52 -8 | <0.001 | 5.71 | 70 | 0.002 |
| Precuneus | L/R | 0 -60 30 | <0.001 | 5.37 | 80 | 0.001 |
| Cerebellum^a^ | L | -12 -76 -28 | <0.001 | 5.04 | 50 | 0.011 |

Brain regions showing a significant increase or decrease in fALFF from pre-encoding rest (R1) to delayed post-encoding rest (R3) in the control condition (CON), indicating consolidation-related activity in the resting state. Clusters are reported at *pFDR* < 0.05. Abbreviations: L, left; R, right; SMG, supramarginal gyrus; MTG, middle temporal gyrus; LG, lingual gyrus.

^a^additional significant cluster compared to the within-mask contrast.

**Supplementary Table 5.** Multiple regression analyses for CON memory performance as the dependent variable (across and within groups)

|  | Predictor Variables | *B* | *SE B* | β | *t* | p-value  (2-tailed) | p-value  (1-tailed) |
| --- | --- | --- | --- | --- | --- | --- | --- |
| HY + HS | mean fALFF CON R1-R3 MTG r | 10.61 | 5.08 | .318 | 2.09 | .047* | .0235* |
|  | FDJenkinson | -1.28 | 41.77 | -.006 | -0.03 | .976 | .488 |
|  | Age | -0.42 | 0.16 | -.520 | -2.69 | .013* | .0065** |
| HY | mean fALFF CON R1-R3 MTG r | 17.15 | 8.07 | .608 | 2.13 | .059 | .0295* |
|  | FDJenkinson | 95.55 | 144.64 | .189 | 0.66 | .524 | .262 |
| HS | mean fALFF CON R1-R3 MTG r | 4.92 | 6.85 | .196 | 0.72 | .486 | .243 |
|  | FDJenkinson | 9.42 | 34.58 | .074 | 0.27 | .790 | .395 |

Resting state-activity changes from pre-encoding rest (R1) to delayed post-encoding rest (R3) in the control condition (CON) significantly predict memory performance irrespective of motion for HY but not for HS. HY, healthy HY participants; HS healthy senior participants; rMTG, right middle temporal gyrus.

Note: * p < .05; ** p < .01; *** p < .001.

**
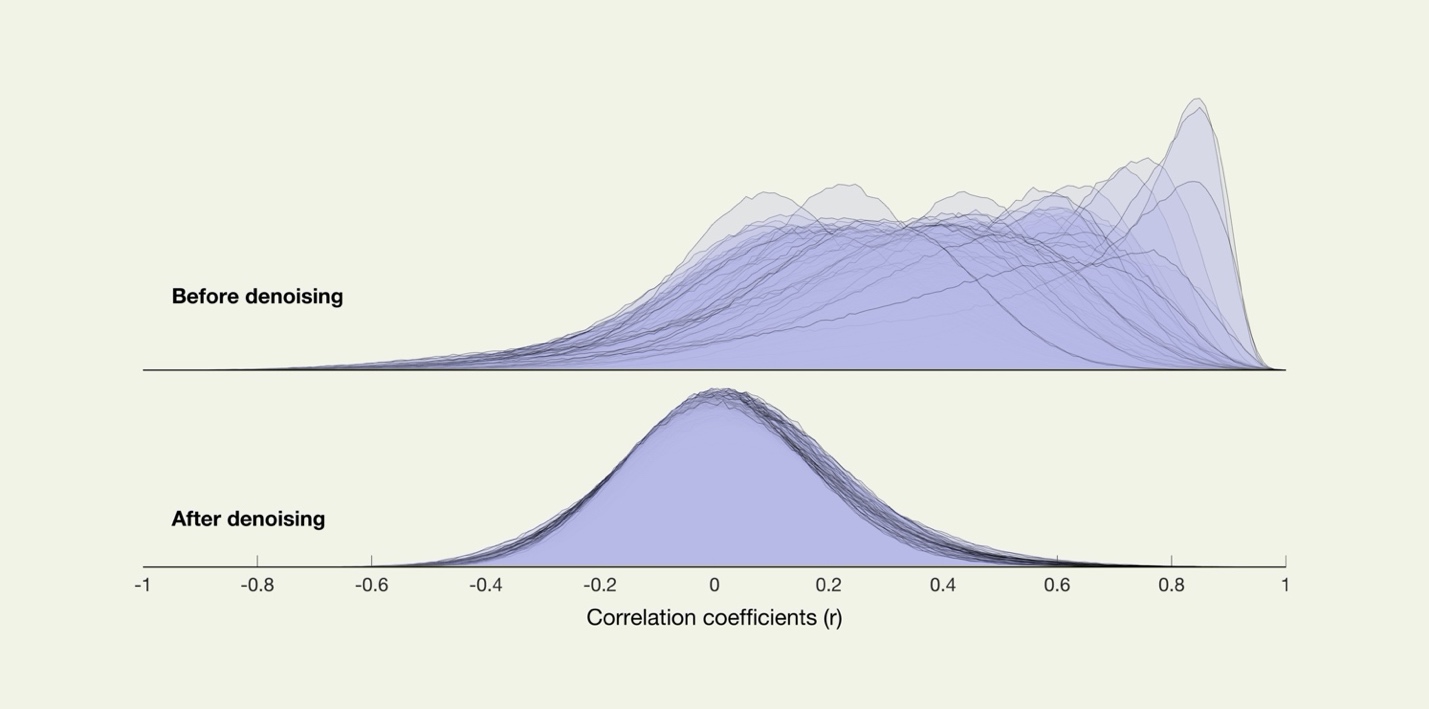
**

**Supplementary Figure 1.** Estimation of the distribution of functional connectivity (FC) values between randomly selected pairs of points in the brain before and after noise reduction.

***
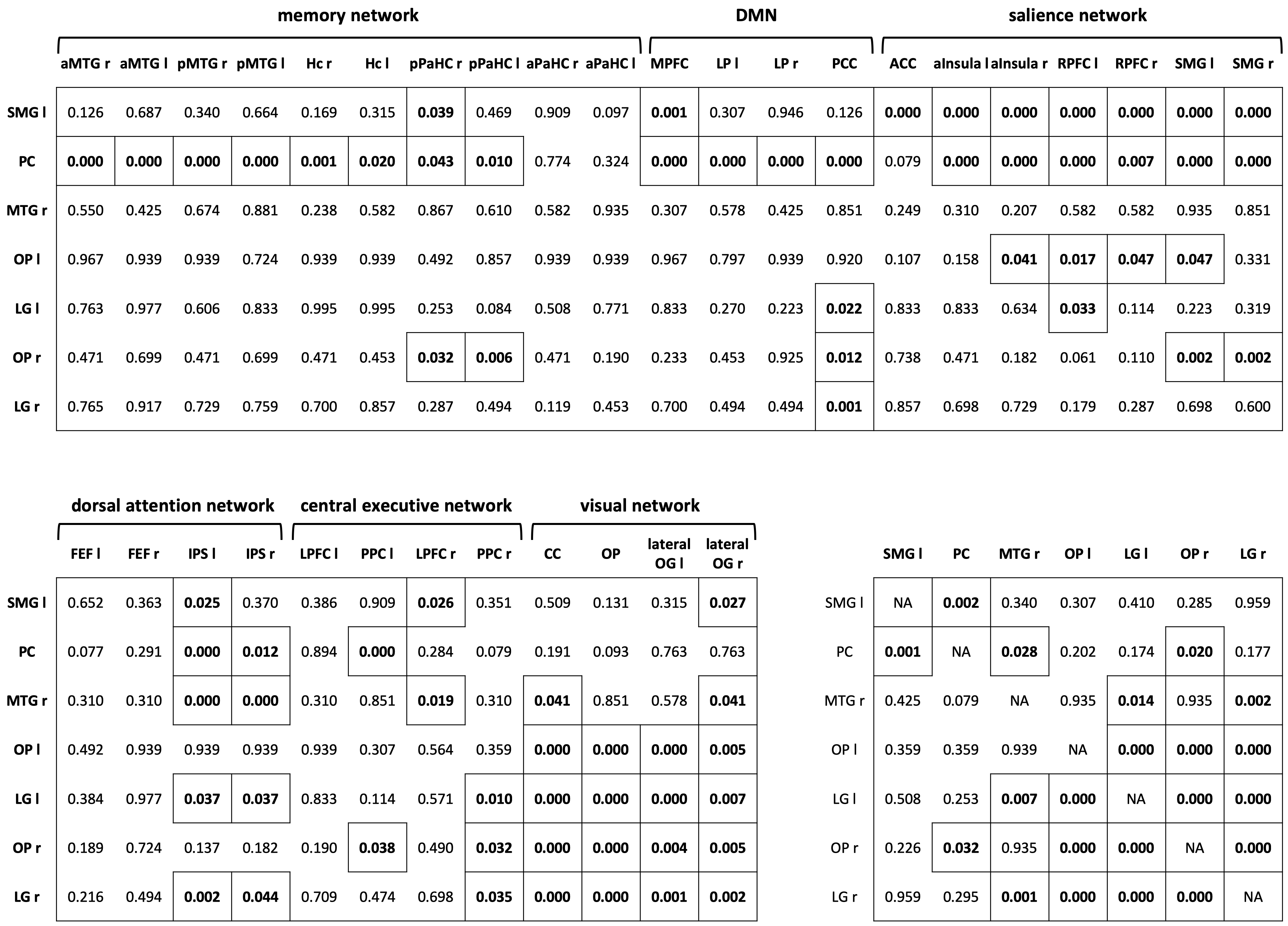
***

**Supplementary Figure 2.** FDR-corrected p-values of one-sample t-tests of all ROI-to-ROI associations of the post-encoding rest session of the control condition. Significant FDR-corrected p-values are bold and outlined (p-FDR<.05). SMG - supramarginal gyrus, PC - precuneus, MTG - middle temporal gyrus, OP - occipital pole, LG - lingual gyrus, aMTG - anterior MTG, pMTG - posterior MTG, Hc - hippocampus, pPaHC - posterior parahippocampal cortex, aPaHC - anterior parahippocampal cortex, MPFC - medial prefrontal cortex, LP - lateral parietal cortex, PCC - posterior cingulate cortex (including the Pc), ACC - anterior cingulate cortex, aInsula - anterior insula, RPFC - rostral prefrontal cortex, FEF - frontal eye field, IPS - inferior parietal cortex, LPFC - lateral prefrontal cortex, PPC - posterior parietal cortex, CC – calcarine cortex, lateral OG - lateral occipital gyrus.
